# Screening biomarkers for predicting the efficacy of immunotherapy in patients with PD-L1 overexpression

**DOI:** 10.1007/s00432-023-05160-9

**Published:** 2023-07-19

**Authors:** Xiaodan Zhu, Bo Yu, Yanli Shen, Yan Zhao, Xiyujing Fu, Yunji Zhu, Guomin Gu, Chunling Liu

**Affiliations:** 1 Department of Pulmonary Medicine, Affiliated Cancer Hospital of Xinjiang Medical University, No. 789 Suzhou East Street, Xinshi District, Urumqi, 830000 Xinjiang China; 2 Department of Medicine, Beijing USCI Medical Laboratory, No. 65, Xingshikou Road, Haidian District, Beijing, 100195 China

**Keywords:** NSCLC, Immunotherapy, PD-L1, Biomarkers

## Abstract

**Purpose:**

Immunotherapy plays an important role in non-small cell lung cancer (NSCLC); in particular, immune checkpoint inhibitors (ICIs) therapy has good therapeutic effects in PD-L1-positive patients. This study aims to screen NSCLC patients with PD-L1-positive expression and select effective biomarkers for ICI immunotherapy.

**Methods:**

Collected tumor samples from the Affiliated Cancer Hospital of Xinjiang Medical University and 117 patients with stage III–IV NSCLC were included in the study. All patients were on first- or second-line therapy and not on targeted therapy. Based on the molecular profiles and clinical features, we screened biomarkers for predicting the efficacy of immunotherapy in patients with PD-L1 overexpression.

**Results:**

117 NSCLC patients receiving ICIs immunotherapy were enrolled. First, we found that immunotherapy was more effective in patients with positive PD-L1 expression. Second, we found that *ROS1* gene mutations, *KRAS* gene mutations, tumor stage, and the endocrine system diseases history are independent prognostic factors for PD-L1 positive patients. Then we combined independent risk factors and constructed a new Nomogram to predict the therapeutic efficacy of ICIs immunotherapy in PD-L1 positive patients. The Nomogram integrates these factors into a prediction model, and the predicted C-statistic of 3 months, 6 months and 12 months are 0.85, 0.84 and 0.85, which represents the high predictive accuracy of the model.

**Conclusions:**

We have established a model that can predict the efficacy of ICIs immunotherapy in PD-L1 positive patients. The model consists of *ROS1* gene mutations, *KRAS* gene mutations, tumor staging, and endocrine system disease history, and has good predictive ability.

**Supplementary Information:**

The online version contains supplementary material available at 10.1007/s00432-023-05160-9.

## Introduction

Lung cancer is the second cancer with the second incidence rate in the world. With an estimated 2.2 million new cases and 1.79 million deaths each year, cancer is one of the most common cancers in the world and the leading cause of cancer-related deaths (Thai et al. 2021). Approximately 80–85% of lung cancer is non-small cell lung cancer (NSCLC) (Sung et al. 2020). In the past decade, personalized treatment of advanced NSCLC has been developing, and evidence of biomarker-based molecular pathways and/or tumor targeting genes is required for specific targeted therapies (Tan and Tan 2022). At the same time, immune checkpoint inhibitors (ICIs) have changed the treatment strategy for NSCLC (Reck et al. 2022). Regardless of previous treatment history, the benefits of ICIs compared to previous standard therapies (cytotoxic chemotherapy) have been demonstrated as both single and combined therapy (Herbst et al. 2016). The reaction duration of ICIs is often longer than that of cytotoxic chemotherapy (Reck et al. 2016). Some patients with advanced NSCLC undergoing ICIs treatment have a survival period of more than 3 years. Notably, the KEYNOTE-024 study showed a 5-year OS rate of 32% (Hellmann et al. 2019). Anti PD-1)/PD-L1 therapy has become the first-line treatment for NSCLC without driver gene mutations (Lahiri et al. 2023). The effects of ICIs were predicted widely by PD-L1 Tumor Proportional Score (TPS). In the phase 2 KEYNOTE-001 trial, the objective response rate (ORR) of pembrolizumab for patients with PD-L1 TPS ≥ 50%, 1–49%, and < 1% NCSLC subgroup was 45%, 17%, and 11%, respectively (Garon et al. 2015). In addition, the high PD-L1 TPS are also associated with survival benefits of pembrolizumab. In the PD-L1 TPS ≥ 50% and ≥ 1% groups of KEYNOTE-024 and 042, pembrolizumab monotherapy was observed to be superior to chemotherapy (Reck et al. 2016; Mok et al. 2019). Subgroup analysis of these studies showed that the higher the PD-L1 TPS, the better the efficacy of ICIs. In the real-world study, this association has been confirmed when limited to PD-L1 TPS ≥ 50% (Aguilar et al. 2019).

The TPS of PD-L1 appeared to be the most commonly used biomarker in the sub analysis of results, which was used as a screening molecular biomarker for first-line monotherapy with pembrolizumab. However, although the efficacy of anti PD-1/PD-L1 drugs is relatively good when considering patients with PD-L1 TPS > 1%, there are still many patients who cannot benefit clinically, which highlights the inadequacy of PD-L1 expression as the sole biomarker, but also highlights the complexity of patient responses to ICIs.

Studies have shown that many clinical factors can also affect the efficacy of immunotherapy. In addition to the PD-L1 TPS, many other clinical factors should also be considered to determine the suitability for ICI immunotherapy, including age, presentation status, histological subtypes, comorbidities, carcinogenic driven mutation status, site of metastasis and so on (Nakagawa and Kawakami 2022). Systemic inflammation was found to cause tumor growth and progression and, therefore, was associated with poor survival in various types of cancer (Möller et al. 1997). For example, changes in the ratio of peripheral blood biomarkers that can reflect this process in patients with malignancy based on changes in lymphocyte numbers [lymphocyte (PLR)] and cytokine levels (Bai et al. 2020). Many studies have shown that immune markers, such as NLR, PLR, and IL-6, are also predictors of the effectiveness of an ICI (Suh et al. 2018; Keegan et al. 2020).

A correlation has also been reported between driver mutation subtypes and ICI efficacy. The ImmunoTarget group retrospectively compared the ORR after ICI treatment in NSCLC patients with various driver mutations. Studies have shown that subgroups of *KRAS* and *BRAF* drivers benefit more from ICI than those of *EGFR* or *ALK* drivers (Mazieres et al. 2019). Although there are many studies on the prognosis of ICIs-based therapy in NSCLC, there are many factors affecting ICI immunotherapy, involving various clinical factors and genetic mutations in patients, especially for patients with PD-L1 TPS > 1. Previous studies have rarely explored the impact of both clinical factors and genetic mutations on ICIs therapy in patients. Therefore, in this retrospective study of NSCLC patients receiving ICIs, we analyzed the clinical characteristics and the impact of genetic mutation situation on the outcome of ICIs therapy and developed a predictive model for ICIs for NSCLC based on these risk factors.

## Materials and methods

### Patients

In this retrospective cohort study, all clinical data were extracted from the medical records of patients with advanced NSCLC who received PD-L1 blocking therapy and platinum chemotherapy in the Cancer Hospital affiliated to Xinjiang Medical University, China, from January 2019 to March 2022. The clinical follow-up ended on May 31, 2022. Eligible patients also meet the following criteria:

I: The patient has a clinical and pathological diagnosis of NSCLC (stage III–IV);
II: Complete clinical information;
III: High expression of PD-L1 and receiving ICIs immunotherapy;
IV: Patients with PD-L1 negative but receiving ICIs immunotherapy served as controls;
V: No other concurrent cancers.

The study was conducted in accordance with the Helsinki Declaration (revised in 2013). The study has been approved by the Ethics Committee of the Cancer Hospital Affiliated to Xinjiang Medical University, and personal consent for this retrospective analysis has been waived.

### Clinicopathological variables

We collected information about gender, age, smoking status, tumor size, tumor subtypes, PD-L1 expression status, previous systemic treatment times, distal metastasis status, family history, tumor stage, respiratory system disease history (RSDH), cardiovascular disease history (CDH), endocrine system disease history (ESDH), and ICIs type. We also recorded triiodothyronine (T3), thyroid stimulating hormone (TSH), thyroxine (T4), adrenocorticotropic hormone (ACTH), troponin, interleukin-2, interleukin-6, interleukin-1 β, interleukin-10, interferon-γ, interleukin-17, interleukin-4, interleukin-12p70, tumor necrosis factor-α, T-helper-induced cells, T-suppressor cytotoxic cells, T cell count, lactate dehydrogenase, creatine kinase isoenzyme, creatine kinase, NL ratio, carcinoembryonic antigen (CEA), carbohydrate antigen 125 (CA125), squamous cell carcinoma antigen (SCC), gastrin releasing peptide precursor (ProGRP), neuron specific enolase (NSE), carbohydrate antigen 199 (CA199), carbohydrate antigen 724 (CA724), white blood cell count (WBC) and whether there are adverse reactions. Based on the treatment response and the evaluation criteria for solid tumor response (RECIST) version 1.1, patients were divided into complete response (CR), partial response (PR), and stable disease (SD) and progressive disease (PD) based on the first CT results after ICI treatment.

### Gene mutation analysis

The NSCLC samples were performed on NGS test with 43 cancer-related genes panel. DNA was extracted from tissue using QIAamp DNA FFPE Tissue Kit (Qiagen, Germany). DNA concentration was estimated using a Qubit fluorometer and Qubit dsDNA High Sensitivity (HS) Assay Kit (Invitrogen, USA). 50–100 ng of sheared genomic DNA was subjected to library construction with an MGIEasy universal DNA library kit (MGI, China), followed by hybrid capture using an xGen Hybridization and Wash Kit (IDT, USA). The qualified libraries were sequenced with 2 × 100 bp paired-end reads on a MGISEQ-2000 (MGI, China) platform.

### Bioinformatics analysis

The paired-end reads were aligned to human reference genome GRCh37/hg19 using BWA-MEM (v0.7.17). SNVs and InDels were called by VarScan (v 2.4.3) by verified settings. SNVs and InDels from tissue were filtered by mean depths > 800×. At least 5 supporting reads were needed for InDels, while 8 supporting reads were needed for SNVs to be called. CNVs were analyzed with in-house algorithm based on sequencing depth of coverage data of capture intervals. The minimum threshold of copy number gain or loss was CN > 2.75 or CN < 1.75 for hotspot genes, and CN > 3 or CN < 1.5 for others. Gene fusion was analyzed using FACTERA.

### Statistical analysis

Clinical and demographic data of patients were analyzed using the Pearson *χ*^2^ test or Fisher’s exact test for categorical variables and the Mann–Whitney *U* test for continuous variables. Univariate logistic regression analysis was used to assess the significance of the impact of each single factor on predicting prognosis of patients treated with ICI, and these significant variables in univariate analysis were used in multivariate analysis to identify potential risk factors for prognosis impact. The odds ratio (OR) and its 95% confidence interval (CI) are presented. Statistical tests were two-sided, with 5% set to the level of significance. All variables with a *p* value < 0.05 in the univariate logistic regression analysis were included in the multivariate analysis to produce an OR and a 95% CI. Kaplan–Meier survival analysis was used to evaluate the association between risk factor and PFS. *p* < 0.05 was considered statistically significant.

## Results

### Participant clinical features

From January 2019 to March 2022, a total of 117 NSCLC patients receiving ICIs immunotherapy were enrolled in this study. Among them, 97 patients showed positive PD-L1 expression (PD-L1 TPS ≥ 1%), 50 patients showed 50% > PD-L1 TPS ≥ 1%, and 47 patients showed PD-L1 TPS ≥ 50%. 20 patients with negative PD-L1 expression (PD-L1 TPS < 1%). In our study, the median age is 62 years old (ranging from 39 to 83 years old). There are 87 (74.4%) males and 30 (25.6%) females. Among the patients, 70 (59.8%) were current smokers or had a history of smoking, and 47 (40.2%) were never smokers. 80 patients (68.4%) were diagnosed with lung adenocarcinoma, 31 patients (26.5%) with lung squamous cell carcinoma, and 6 patients (5.1%) with other subtypes. In this cochort, 35 (29.9%) patients were in stage III and 82 (70.1%) patients were in stage IV. There were 79 (67.5%) patients with distant metastasis, 31 (26.5) patients without distant metastasis, and 7 (6.0%) undefined samples. Among them, 63 (53.8%) patients received first-line immunotherapy, and 54 (46.2%) patients received non-first-line immunotherapy. In addition, we also found that a total of 75 patients had a data of history of endocrine system diseases, of which 16 had a history of endocrine system diseases, 59 had no history of endocrine system diseases, and 42 people were not counted for the relevant information. We found that a total of 97 patients had data on white blood cell counts, with a median of 6.35 × 10^9^/L (2.97 × 10^9^/L–15.87 × 10^9^/L) (Table 1). In addition, other clinical information can be found in the supplementary materials. (Supplementary Table 1).

**Table 1:** Summary of baseline patient characteristics

	Cohort (*n* = 117)	PD-L1 expression ≥ 50% (*n* = 47)	50% > PD-L1 expression ≥ 1% (*n* = 50)	PD-L1 expression < 1% (*n* = 20)	*p* value
Median age (range)	62 (39–83)	66 (44–81)	61 (39–83)	63.5 (45–76)	
< 63	55 (47.0%)	20 (42.6%)	31 (62.0%)	10 (50.0%)	
≥ 63	62 (53.0%)	27 (57.4%)	19 (38.0%)	10 (50.0%)	
Gender					
Male	87 (74.4%)	38 (80.9%)	34 (68.0%)	15 (75.0%)	
Female	30 (25.6%)	9 (19.1%)	16 (32.0%)	5 (25.0%)	
Smoking history					
Smoking	70 (59.8%)	28 (59.6%)	29 (58.0%)	13 (65.0%)	
No smoking	47 (40.2%)	19 (40.4%)	21 (42.0%)	7 (35.0%)	
Histology					
Adenocarcinoma	80 (68.4%)	34 (72.3%)	34 (68.0%)	12 (60.0%)	
Squamous cell carcinoma	31 (26.5%)	9 (19.1%)	14 (28.0%)	8 (40.0%)	
Others	6 (5.1%)	4 (8.6%)	2 (4.0%)	0 (0%)	
TNM					
III	35 (29.9%)	14 (37.8%)	14 (28.0%)	7 (35.0%)	
IV	82 (70.1%)	33 (62.2%)	36 (72.0%)	13 (65.0%)	
Therapy					
First line	63 (53.8%)	30 (63.8%)	25 (50.0%)	8 (40.0%)	
Non-first line	54 (46.2%)	17 (36.2%)	25 (50.0%)	12 (60.0%)	
Best efficacy					
CR	2 (1.7%)	1 (2.1%)	1 (2.0%)	0 (0.0%)	
PR	43 (36.8%)	28 (59.6%)	10 (20.0%)	5 (25.0%)	
SD	49 (41.9%)	13 (27.7%)	27 (54.0%)	9 (45.0%)	
PD	19 (16.2%)	5 (10.6%)	9 (18.0%)	5 (25.0%)	
NA	4 (3.4%)	0 (0.0%)	3 (6.0%)	1 (5.0%)	
History of endocrine system diseases					
Yes	16 (13.7%)	8 (17.0%)	6 (12.0%)	2 (10.0%)	
No	59 (50.4%)	24 (51.1%)	31 (62.0%)	4 (20.0%)	
Unknown	42 (35.9%)	15 (31.9%)	13 (26.0%)	14 (70.0%)	
WBC					
≥ 6.35	49 (41.9%)	24 (51.1%)	28 (56.0%)	2 (10.0%)	
< 6.35	48 (41.0%)	18 (38.3%)	19 (38.0%)	0.6 (30.0%)	
NA	20 (17.1%)	5 (10.6%)	3 (6.0%)	12 (60.0%)	

### Survival analysis

**Fig. 1:**
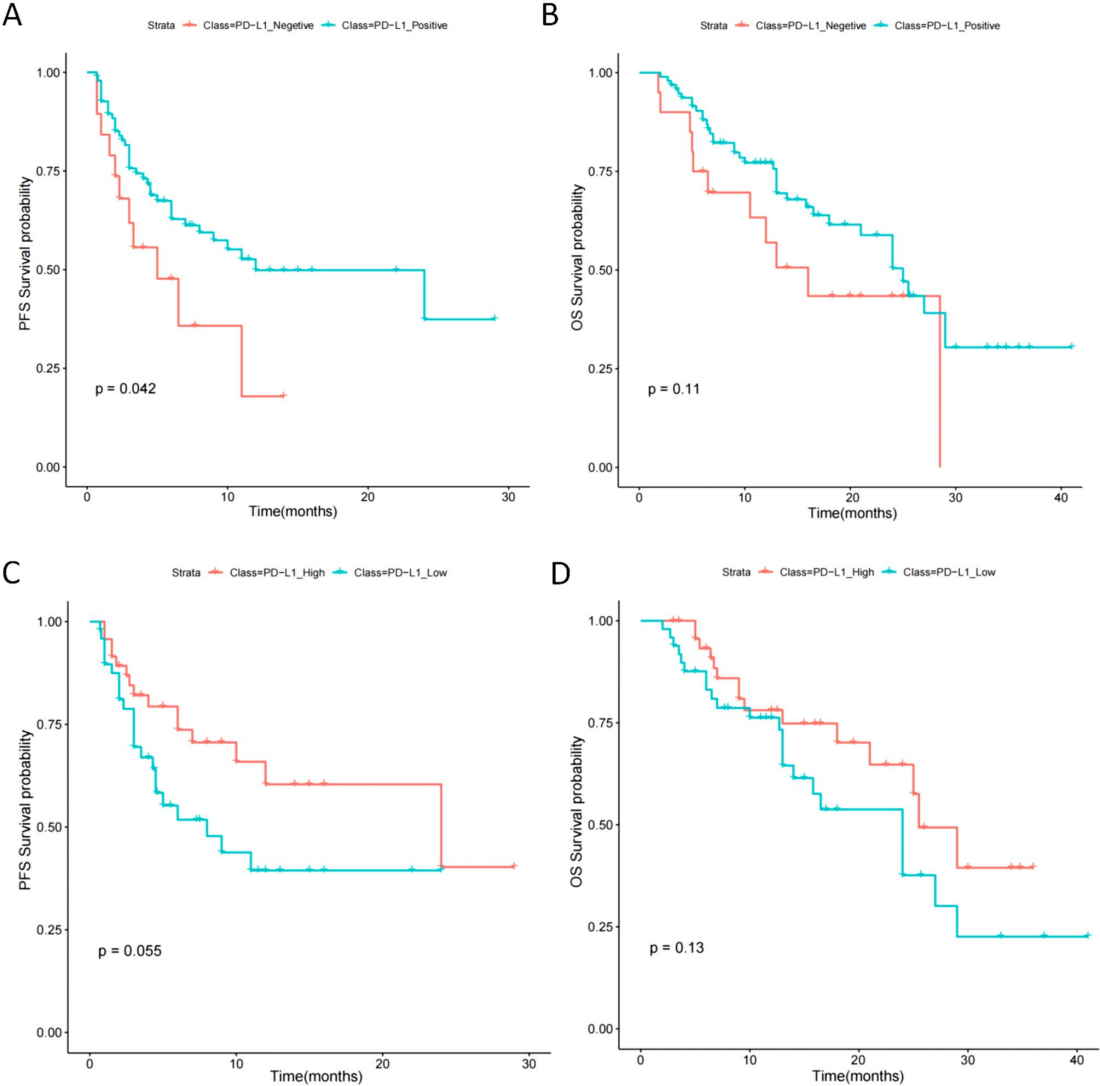
Kaplan–Meier survival curve to compare PFS and OS analysis between patients with PD-L1 expression positive (PD-L1 TPS ≥ 1%) and patients with PD-L1 expression negative (PD-L1 TPS < 1%). **A** The PFS between patients with positive PD-L1 expression and patients with negative PD-L1 expression. **B** The OS between patients with positive PD-L1 expression and patients with negative PD-L1 expression. **C** The Kaplan–Meier survival curve of PFS in patients with PD-L1 TPS ≥ 50% (PD-L1 high) compared to those with 50% > PD-L1 TPS ≥ 1% (PD-L1 low). **D** The Kaplan–Meier survival curve of OS in patients with PD-L1 TPS ≥ 50% (PD-L1 high) compared to those with 50% > PD-L1 TPS ≥ 1% (PD-L1 low)

To validate the ability of PD-L1 as a predictive marker for ICIs immunotherapy, we constructed a Kaplan–Meier survival curve to compare PFS and OS analysis between patients with PD-L1 expression positive (PD-L1 TPS ≥ 1%) and patients with PD-L1 expression negative (PD-L1 TPS < 1%). The results showed that there was a significant difference in PFS between patients with positive PD-L1 expression and patients with negative PD-L1 expression (Fig. 1A), but there was no significant difference in OS (Fig. 1B). Furthermore, we analyzed the survival differences between patients with PD-L1 expression positive (PD-L1 TPS ≥ 1%) and found that although PFS and OS were slightly improved in patients with PD-L1 TPS ≥ 50% compared to those with 50% > PD-L1 TPS ≥ 1%, there was no significant difference (Fig. 1C, D). So in order to explore which patients are more suitable for ICIs immunotherapy among PD-L1 expression positive patients, it may be necessary to add other biomarkers for judgment.

### Prognostic-related clinical factors in patients with positive PD-L1 expression

To evaluate the clinical factors that affect the therapeutic efficacy of PD-L1 positive ICI immunotherapy, we conducted a univariate COX regression analysis on the collected clinical information of patients, and the results showed that clinical stage, presence of distant metastasis, endocrine system diseases history, whether immunotherapy is first-line treatment, and white blood cell count can significantly affect PFS. Tumor stage (OR 4.51; 95% CI 1.69–14.97, *p* = 0.014), presence of distant metastasis (OR 7.17; 95% CI 1.69–30.32, *p* = 0.007), endocrine system diseases history (OR, 2.66; 95% CI 1.08–6.53, *p* = 0.033), and white blood cell count levels (OR 1.17; 95% CI 1.01–1.34, *p* = 0.032) are associated with an increased risk of poor prognosis. The first-line use of ICIs immunotherapy (OR 0.44; 95% CI 0.21–0.92, *p* = 0.03) may have better therapeutic effects (Table 2).

**Table 2:** Univariate COX regression analysis of potential risk factors for Immunotherapy prognosis

Characteristics		HR	95% CI		*p* value
			Lower	Upper	
Tumor stage	IV vs. III	4.51	1.36	14.97	0.014
Distal metastasis	Yes vs. No	7.17	1.69	30.32	0.007
History of endocrine system diseases	Yes vs. No	2.66	1.08	6.53	0.033
WBC	≥ 6.35 vs. < 6.35	1.17	1.01	1.34	0.032
Treatment lines	First vs. non-first	0.44	0.21	0.92	0.030
ROS1	Mutation vs. WT	1.39	1.15	2.05	0.042
KRAS	Mutation vs. WT	0.76	0.36	0.96	0.009
ERBB2	Mutation vs. WT	1.88	1.33	3.59	0.031

### Analysis of gene mutations

**Fig. 2:**
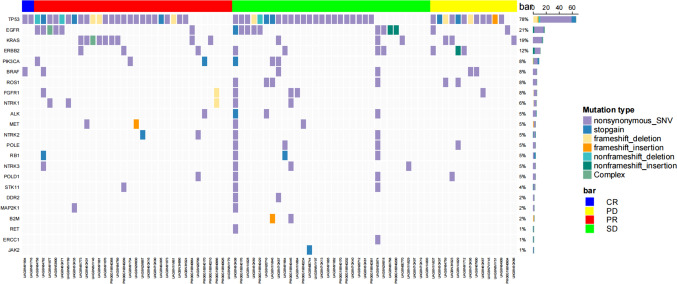
Gene mutation profile of PD-L1 positive patients

In order to investigate gene mutations in PD-L1-positive patients, we analyzed the gene mutations in PD-L1-positive patients among the enrolled patients. A panel consisting of 43 lung cancer-related genes was used to sequence the PD-L1-positive samples to detect the incidence of gene mutations. The results showed that out of the 80 patient samples, a total of 74 patients had genetic mutations, with a mutation incidence rate of 92.5%. In advanced NSCLC with positive PD-L1 expression, TP53 has the highest mutation frequency at 78% (62/80), followed by *EGFR*, *KRAS*, and *ERBB2* with higher mutation frequencies at 21% (17/80), 19% (15/80), and 12% (10/80), respectively (Fig. 2).

### Prognostic-related mutated genes in patients with positive PD-L1 expression

**Fig. 3:**
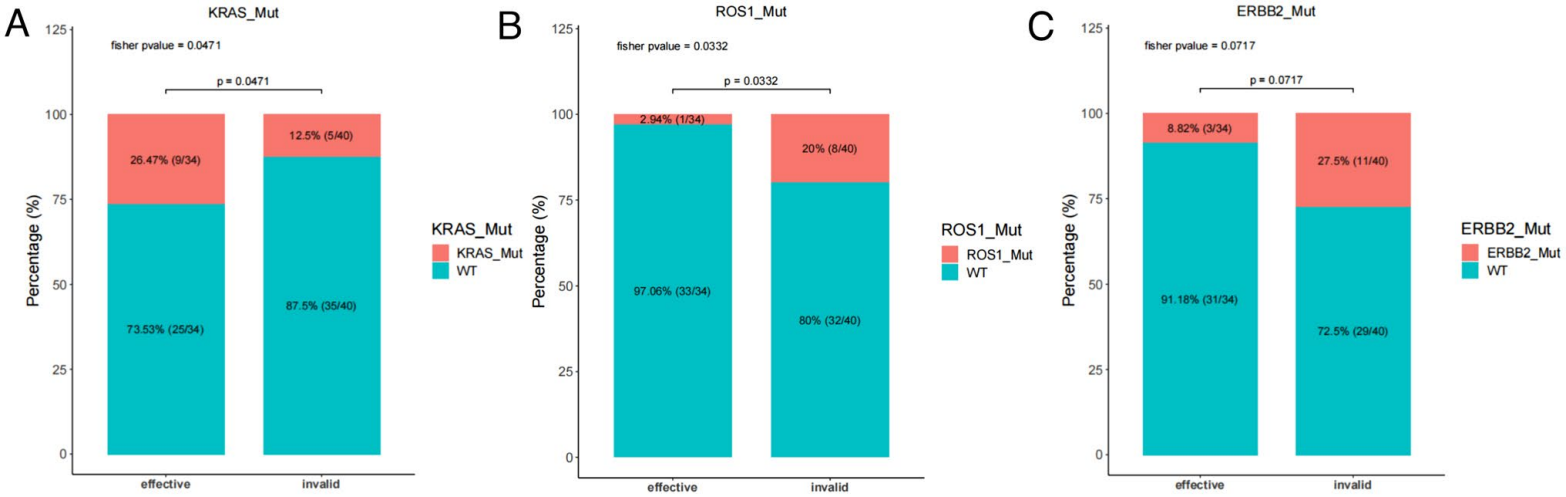
Different mutation gene analysis in patients with positive PD-L1 expression based on immunotherapy efficacy. **A** *KRAS* mutation. **B** *ROS1* mutation. **C** *ERBB2* mutation

Firstly, we found that patients with positive PD-L1 expression had an objective response rate (ORR) of 46.2% and a (Disease Control Rate) DCR of 85.1% through ICI immunotherapy (Supplementary Table 1). In order to identify therapeutic effects-related mutant genes in patients with positive PD-L1 expression, we analyzed the differences in mutated genes between samples with effective response (PR + CR) to ICIs treatment and those without effective response (SD + PD). The results showed significant differences in *KRAS* (*p* = 0.0471) and *ROS1* (*p* = 0.0332) mutations. The *KRAS* gene mutation is mainly present in patients who have response through ICIs immunotherapy (9/34) (Fig. 3A), while the *ROS1* gene mutation is mainly present in stable and progressive populations with the best treatment effect (8/40) (Fig. 3B). In addition, we found that ERBB2 gene mutations mainly occurred in the SD and PD populations (11/40), with only 3 cases (3/34) of ERBB2 mutations present in the CR and PR populations, but there was no significant difference between the two (*p* = 0.0717) (Fig. 3C). In order to better identify mutated genes that affect ICIs immunotherapy, we also conducted univariate COX regression analysis to identify mutated genes related to prognosis. The results showed that *ROS1, KRAS*, and *ERBB2* were correlated with PFS (Table 2).

### Nomogram construction and validation

**Fig. 4:**
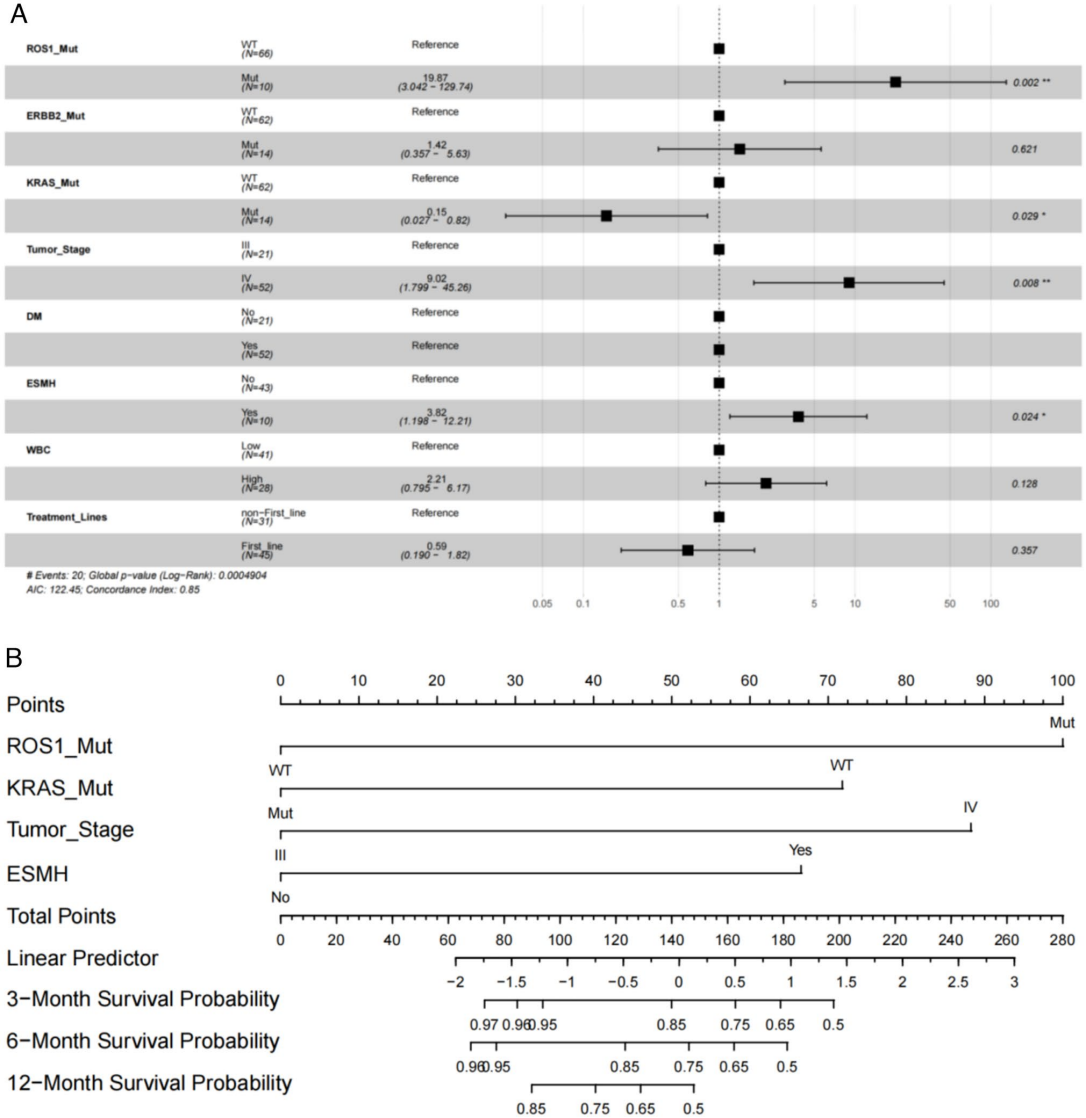
Multivariate COX regression analysis and Nomogram construction. **A** Multivariate COX regression analysis was conducted on clinical factors and mutated genes. **B** The Nomogram model that including *ROS1* gene mutations, *KRAS* gene mutations, tumor stage, and endocrine system disease history

Multivariate COX regression analysis was conducted on clinical factors and mutated genes which related to prognosis, to further determine the relevant factors for ICIs immunotherapy in PD-L1 positive patients. The results showed that *ROS1* gene mutations, tumor stage, and the endocrine system diseases history were independent adverse prognostic factors, while *KRAS* gene mutations were independent prognostic protective factors (Fig. 4A).

Combining independent risk factors from multivariate analysis, including *ROS1* gene mutations, *KRAS* gene mutations, tumor stage, and endocrine system disease history, a new Nomogram was constructed to predict the therapeutic efficacy of ICIs immunotherapy in PD-L1 positive patients. The Nomogram model intuitively reveals the important contributions of *ROS1* gene mutations, *KRAS* gene mutations, tumor staging, and endocrine system disease history to prognosis prediction (Fig. 4B). The Nomogram integrates these factors into a prediction model, and the predicted C-statistic of 3 months, 6 months and 12 months are 0.85 (95% CI 0.77–0.94), 0.84 (95% CI 0.73–0.96) and 0.85 (95% CI 0.68–0.99) which represents the high predictive accuracy of the model.

We studied the correlation of the prediction model consisting of *ROS1* gene mutations, *KRAS* gene mutations, tumor stage, and endocrine system disease history with PD-L1 expression, and we assessed whether the prediction model was an independent risk factor for survival prediction by the multivariable Cox regression analysis. The result showed that the prediction model risk score was an independent prognostic factor after adjusting PD-L1 expression (Supplementary Table 2).

**Fig. 5:**
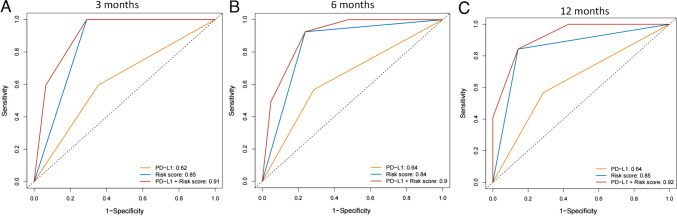
The ROC curve of the prediction model. **A** The ROC curve of the prediction model that predict the 3 months of PFS. **B** The ROC curve of the prediction model that predict the 6 months of PFS. **C** The ROC curve of the prediction model that predict the 12 months of PFS

Next, we constructed the ROC curve of the prediction model and found that the area under the ROC curve (AUC) value for 3 months. 6 months and 12 months are 0.85 (95% CI 0.77–0.94), 0.84 (95% CI 0.73–0.96) and 0.85 (95% CI 0.68–0.99), respectively (Fig. 5A–C). This indicated a higher prediction performance. When compared with PD-L1 expression as risk prediction, it was found that our model had better predictive performance than PD-L1 (Fig. 5A–C). When our model added PD-L1 expression, the combined predictive model had better predictive performance. The AUC value for 3 months, 6 months and 12 months is 0.91 (95% CI 0.83–0.99), 0.90 (95% CI 0.81–0.99) and 0.92 (95% CI 0.82–0.99), respectively (Fig. 5A–C).

### Model prediction performance verification

**Fig. 6:**
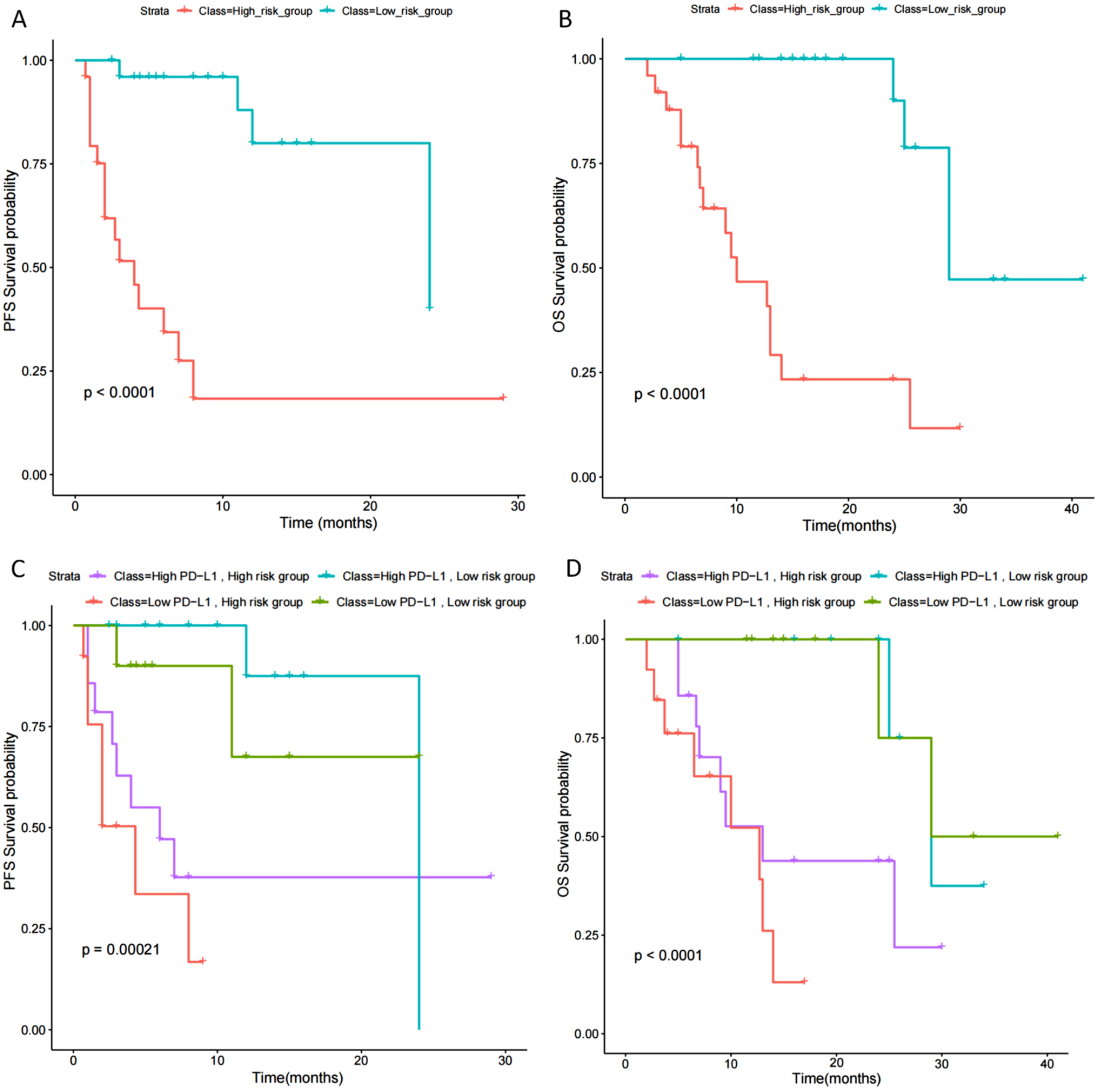
Stratified analysis of high-risk score and low-risk score of the prediction model. **A** The Kaplan–Meier survival curve of PFS for the high-risk and low-risk people. **B** The Kaplan–Meier survival curve of OS for the high-risk and low-risk people. **C** The Kaplan–-Meier survival curve of PFS for the risk score combined the PD-L1 expression. **D** The Kaplan–Meier survival curve of OS for the risk score combined the PD-L1 expression

To test the predictive performance of the model, we constructed a Kaplan–Meier survival curve to test the model's predictive prognostic ability. We divided the group into low-risk and high-risk groups based on the median risk score (cut-off value = 7.5). We observed that patients in the high-risk group had significantly shorter PFS and OS after ICIs immunotherapy compared to the low-risk group (the mean high-risk group PFS VS. the mean low-risk group PFS: 4.256 months vs. 9.832 months) (Fig. 6A, B, Supplementary Table 2). When our model was combined with PD-L1 grouping, it was found that patients with PD-L1 TPS ≥ 50% in the low-risk population had the best survival (the mean PFS is 10.63 months), while patients with PD-L1 TPS ≥ 1% in the high-risk group had poorer prognosis (the mean PFS is 2.39 months) (Fig. 6C, D, Supplementary Table 2).

## Discussion

Immune checkpoint inhibitors, such as PD-1 and PD-L1 inhibitors, have completely changed the treatment of many cancers, including NSCLC, resulting in improved treatment outcomes for patients, especially those with positive PD-L1 expression who benefit more than those with negative PD-L1 expression (Sharma et al. 2023). However, among PD-L1 positive patients, there are still some patients who cannot benefit, so how to select the most likely patients to benefit from immunotherapy is currently the main challenge in this field. Previous studies have paid little attention to markers of immunotherapy benefits in PD-L1 positive patients, and we have developed and validated a non-invasive and clinically applicable model that combines clinical factors and gene mutation characteristics before treatment to predict the treatment benefits of ICIs immunotherapy in advanced NSCLC PD-L1 positive patients. Here, we identified four factors, including *ROS1* gene mutations, *KRAS* gene mutations, tumor staging, and a history of endocrine system diseases, which are associated with treatment efficacy in patients with PD-L1 positive expression. And we found that this model has good predictive ability.

*ROS1* is an oncogene encoding receptor tyrosine kinase, which shows considerable homology with other members of receptor tyrosine kinase insulin receptor family, especially ALK (Priest et al. 2023). A previous study showed that high PD-L1 expression (PD-L1 TPS ≥ 50%) in late stage non-small cell lung cancer with negative driver mutations predicted a good response to ICIs monotherapy (Reck et al. 2016). In addition, in the immunohistochemical registration study, the total effective rate of ROS1 fusion NSCLC patients was 16.7%, which was unsatisfactory (Schoenfeld et al. 2019). In our study, we also found that ROS1 gene mutations mainly occurred in populations with poor treatment efficacy. We mainly tested gene mutations in 74 patients and found that 9 patients had ROS1 mutations, with the main variant subtype being ROSI fusion. Out of 9 patients, 8 had the best treatment outcome for SD or PD, with only 1 patient receiving effective relief after treatment. And we also found that *ROS1* gene mutation is a risk factor for predicting the efficacy of ICIs immunotherapy.

The *KRAS* gene has been proven to be one of the most common carcinogenic drivers of human cancer (O'Sullivan et al. 2023). Over the years, there have been many studies on the relationship between *KRAS* gene mutations and the efficacy of immunotherapy (Li et al. 2022; Liu et al. 2022). A meta-analysis by Lee et al. (2018) reported that compared to docetaxel, ICIs appeared to improve survival in the *KRAS* gene mutant patient subgroup, but not in *KRAS* wild type patients. A subgroup analysis of another randomized phase III study from CheckeMate057, showed that during the second-line treatment period of *KRAS* gene mutation patients, Nilumab monotherapy had a higher OS benefit than docetaxel monotherapy (Borghaei et al. 2015). The OAK study is a randomized, double-blind phase III study. Based on the *KRAS* mutation status, OS analysis results indicate that NSCLC patients with *KRAS* mutations may also benefit from azozumab in terms of OS (Rittmeyer et al. 2017). Our study also found that *KRAS* gene mutation is a protective factor for ICIs immunotherapy, which is consistent with previous studies and confirms the viewpoint that *KRAS* gene mutation is beneficial for immunotherapy.

In our study, we also found that tumor stage and the endocrine system diseases history have a significant impact on ICIs immunotherapy. In previous studies, tumor stage had always been a poor prognosis factor, and the higher the stage, the worse the prognosis, which has a significant impact on the therapeutic effect (Remon et al. 2019; Patel and West 2020). In our study, it is also fully demonstrated that although all cases are advanced NSCLC, the prognosis of stage IV is significantly worse than that of stage III, and the therapeutic effect of ICIs is not good. There is relatively little research on the impact of the endocrine system diseases history on immunotherapy, but the endocrine system has a greater impact on immune function. Adrenocorticotropic hormone-releasing hormone can directly promote the production of corticotropin and endorphin by human peripheral white blood cells (after endotoxin pretreatment). Adrenocorticotropic hormone has the effect of inhibiting immune response (Wright and Hayes 2023), and glucocorticoids generally also have the effect of inhibiting immune response (Tedeschi et al. 2023; Huffman et al. 2023). Estrogen, progesterone, and androgen all have inhibitory effects on immune function. Thyrotropin-releasing hormone, thyrotropin and thyroid hormone all have the effect of enhancing immune function (Castellanos et al. 2023; Chang et al. 2023; Yang et al. 2022). Therefore, we can see the impact of endocrine system diseases on immunotherapy.

Overall, we identified an effective predictive model for immunotherapy efficacy in PD-L1 positive patients. However, there are also certain limitations. Firstly, we are a single center study and do not have any other data to validate our model. Secondly, our sample size is relatively small, and we may need a larger sample size for further research in the future. Finally, due to the short follow-up time, our main observation target is PFS, but further observation may be needed for overall survival time.

## Conclusions

We have established a model that can predict the efficacy of ICIs immunotherapy in PD-L1 positive patients. The model consists of *ROS1* gene mutations, *KRAS* gene mutations, tumor staging, and endocrine system disease history, and has good predictive ability. When combined with PD-L1 expression score, the predictive ability is stronger.

## Supplementary Information

Below is the link to the electronic supplementary material.

Supplementary file1 (XLSX 38 KB)

Supplementary file2 (XLSX 21 KB)

## Data Availability

The data used to support the findings of this study are available from the corresponding author upon reasonable request.
